# Purulent Pericarditis Leading to Constriction

**DOI:** 10.14740/cr356w

**Published:** 2014-12-04

**Authors:** Akira Wada, Jason Craft, Ernest L. Mazzaferri

**Affiliations:** a Department of Cardiovascular Medicine, The Ohio State University Wexner Medical Center, Columbus, OH 43210, USA

**Keywords:** Purulent pericarditis, Bacterial pericarditis, Constrictive pericarditis

## Abstract

We report a case of a previously healthy 61-year-old immunocompetent male who was found to have purulent bacterial pericarditis. The patient was initially diagnosed with pneumococcal pneumonia and bacteremia after presenting with chest pain and a productive cough. He was found to have a purulent pericardial effusion and underwent surgical washout and creation of a pericardial window. In short time he developed signs of right heart failure and a cardiac MRI revealed a severely thickened pericardium with evidence of constrictive pericarditis. The patient subsequently underwent pericardiectomy where the diagnosis of constriction was confirmed. Our patient recovered well and had no clinical evidence of heart failure on follow-up. This case demonstrates the importance of rapid identification of bacterial pericarditis and the high likelihood of progression to constriction.

## Introduction

Bacterial pericarditis is a rare but lethal disease with 100% mortality in those who are untreated [1]. The low incidence of bacterial pericarditis is likely accounted for by the wide spread availability of effective antibiotics. This form of pericarditis must be recognized early as this disease entity carries significant morbidity and mortality even with treatment [2].

## Case Report

**Figure 1:**
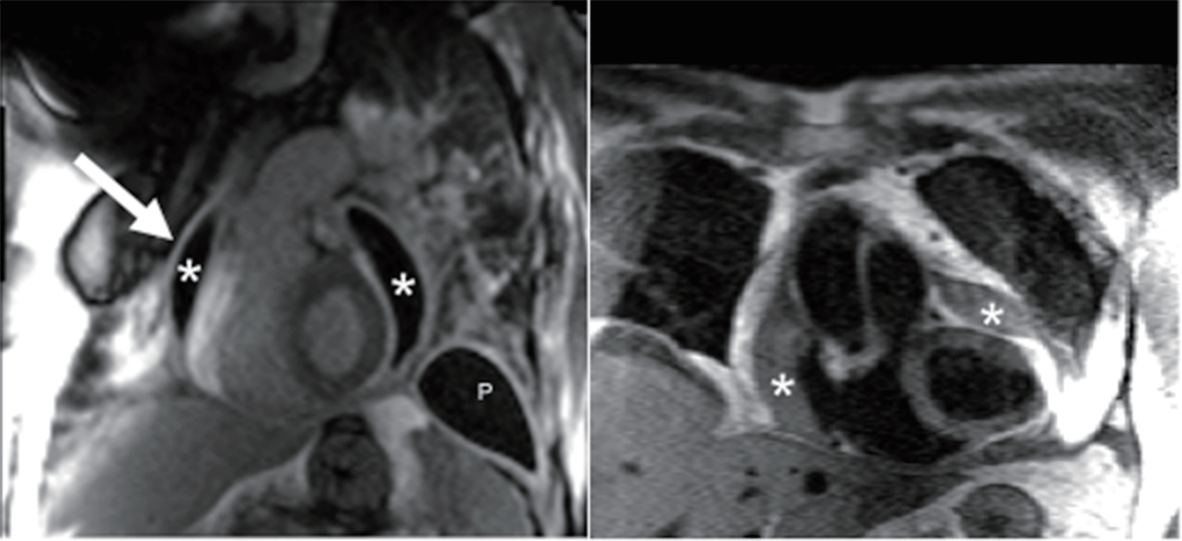
Post-gadolinium inversion recovery sequence with steady state free precession readout demonstrates a grossly thickened pericardium (arrow) accompanied by a loculated pericardial effusion (*). Pleural thickening with effusion (P) is also present. Enhancement of the pleura and pericardium is consistent with significant fibrosis. Double inversion T1-weighted turbo spin echo sequence also demonstrates the findings of a loculated pericardial effusion.

**Figure 2:**
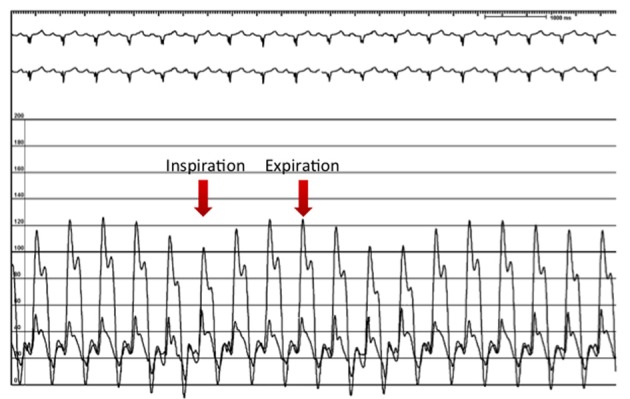
Simultaneous right and left ventricular recordings showing discordant ventricular filling during inspiration, consistent with constrictive physiology.

**Figure 3:**
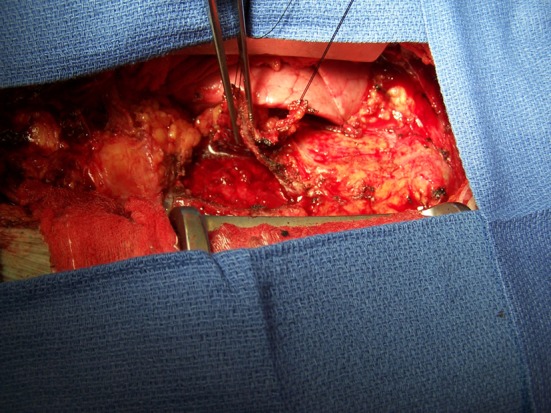
Intraoperative photo reveals a severely thickened parietal pericardium.

We report a case of a previously healthy 61-year-old immunocompetent male who was found to have purulent bacterial pericarditis. The patient had developed a productive cough, shortness of breath and pleuritic chest pain several days prior to presenting to a local emergency department. His initial echocardiogram (ECG) in the emergency department showed diffuse ST segment elevations. He was emergently transferred to our medical center as a possible ST segment myocardial infarction. He underwent emergent left heart catheterization upon arrival, which showed no obstructive coronary artery disease. The patient was tachycardic and hypoxic with an oxygen saturation of 88%. A CT pulmonary angiogram was obtained which showed no acute pulmonary embolism but did reveal a dense left lower lobe consolidation. His WBC upon admission was 18,000/μL with neutrophil predominance (91.1%). C-reactive protein and erythrocyte sedimentary rate were both extremely elevated at 429.8 mg/L (normal < 10) and 123 mm/h (normal < 20), respectively. His labs also revealed a significantly elevated BUN of 103 mg/dL and a creatinine of 4.96 mg/dL. Blood and sputum cultures were obtained, and he was started on ceftriaxone and azithromycin for community acquired pneumonia. Blood cultures returned positive for *Streptococcal pneumoniae* (pan-sensitive) within less than 24 h. His respiratory viral PCR assay was also positive for influenza A (H1N1 subtype), and he was started on oseltamivir. The patient’s WBC continued to rise despite antibiotic and antiviral therapy. A repeat CT of the chest was obtained which re-demonstrated a left lower lobe consolidation and small pleural effusion. A transthoracic ECG was obtained which showed a large circumferential pericardial effusion with right ventricular diastolic collapse and respiratory variation in the mitral and tricuspid inflow velocities. He underwent emergent pericardiocentesis with removal of approximately 350 mL of straw colored fluid. Gram stain of the fluid revealed gram-positive cocci with a WBC of 101,000/μL (99% PMNs), pH 7.01, glucose < 10 mg/dL, LDH 42,550 U/L and protein 4,753 mg/dL. The patient’s WBC count continued to remain elevated post-pericardiocentesis. He underwent surgical drainage and creation of a pericardial window on hospital day 8 due to persistence of the pericardial effusion. The pericardium was thickened and inflamed with a significant amount of necrotic and purulent debris. The necrotic and inflamed debris was manually removed, and a washout was performed with saline. The patient improved significantly post-procedure, and his WBC and creatinine decreased to near normal by discharge. He was continued on ceftriaxone and discharged to a rehabilitation facility after 18 days. He was readmitted to our hospital 11 days after discharge with worsening lower extremity edema and an elevated B-type natriuretic peptide of 439 pg/mL (normal 0 - 100). A cardiac MRI was performed and showed a markedly thickened pericardium with evidence of constriction but preserved biventricular systolic function (Fig. 1). He underwent a simultaneous left and right heart catheterization to evaluate for constrictive physiology. There were elevated and near equalization of diastolic filling pressures (24 mm Hg) in each cardiac chamber, a “dip and plateau” pattern of left ventricular filling, and discordant changes in right and left ventricular systolic pressures with inspiration (Fig. 2). Given these findings, he underwent a pericardiectomy, which revealed a very thickened (2 cm) parietal component of the pericardium. Once the visceral pericardium was dissected, the heart budged out (Fig. 3), confirming the diagnosis of constriction. The patient clinically improved after his pericardiectomy and was discharged. In follow-up the patient had no clinical heart failure symptoms, and his ECG showed normal left and right ventricular function with no evidence of constriction.

## Discussion

Purulent pericarditis has become a rare, but still often fatal disease. Infection usually arises from contiguous spread from the lung or head/neck as well as through hematogenous seeding. The most common organisms implicated are *Staphylococcus*, *Streptococcus*, and *Mycobacterium* species [3]. Factors such as immunodeficiency, chronic inflammatory states, chest trauma or cardiac surgery can predispose individuals to infection. The disease is acute and patients typically present with a rapidly progressive infectious illness. Patients with signs of sepsis should be hospitalized and monitored, ideally in an intensive care unit.

Prompt identification is essential and requires percutaneous pericardiocentesis with gram stain and culture of the pericardial fluid. Rinsing of the pericardial cavity is critical, but open drainage through a surgical pericardial window is preferable. Currently there is no evidence to support the use of intrapericardial fibrinolysis in cases of purulent pericarditis. Systemic antibiotic coverage should include antistaphylococcal agents, and antibiotic therapy should be tailored based on culture results [1]. For patients who develop purulent pericarditis from a contiguous pneumonia (community acquired), a third generation cephalosporin such as ceftriaxone or cefotaximine is recommended for empiric therapy. In patients who present with severe sepsis, the addition of vancomycin is appropriate. For patients who developed pericardial infection secondary to a head and neck source, anerobic coverage is required with either augmentin/sulbactam or a carbapenem such as imipenem [3].

Pericardial constriction is a common complication of purulent pericarditis, and these patients should undergo pericardiectomy [4]. Constrictive pericarditis is caused when the heart is encased in a rigid and dense pericardium due to fibrosis and adhesions. This causes impaired left and right ventricular filling and can lead to heart failure with peripheral edema, hepatic congestion, as well as ascites. Pericardiectomy with biopsy is the definitive measure to secure a diagnosis and for treatment. The procedure has a mortality rate of 6-12% with complete normalization of cardiac hemodynamics in reported only 60% of patients [5, 6].

## References

[1] Maisch B, Seferovic PM, Ristic AD, Erbel R, Rienmuller R, Adler Y, Tomkowski WZ (2004). Guidelines on the diagnosis and management of pericardial diseases executive summary; The Task force on the diagnosis and management of pericardial diseases of the European society of cardiology. Eur Heart J.

[2] Sagrista-Sauleda J, Barrabes JA, Permanyer-Miralda G, Soler-Soler J (1993). Purulent pericarditis: review of a 20-year experience in a general hospital. J Am Coll Cardiol.

[3] Goodman LJ (2000). Purulent Pericarditis. Curr Treat Options Cardiovasc Med.

[4] Bertog SC, Thambidorai SK, Parakh K, Schoenhagen P, Ozduran V, Houghtaling PL, Lytle BW (2004). Constrictive pericarditis: etiology and cause-specific survival after pericardiectomy. J Am Coll Cardiol.

[5] Ling LH, Oh JK, Schaff HV, Danielson GK, Mahoney DW, Seward JB, Tajik AJ (1999). Constrictive pericarditis in the modern era: evolving clinical spectrum and impact on outcome after pericardiectomy. Circulation.

[6] Yetkin U, Kestelli M, Yilik L, Ergunes K, Kanlioglu N, Emrecan B, Ozbek C (2003). Recent surgical experience in chronic constrictive pericarditis. Tex Heart Inst J.

